# Premature cardiovascular disease mortality with overweight and obesity as a risk factor: estimating excess mortality in the United States during the COVID-19 pandemic

**DOI:** 10.1038/s41366-023-01263-y

**Published:** 2023-02-02

**Authors:** Tim Adair

**Affiliations:** The Nossal Institute for Global Health, Melbourne School of Population and Global Health, The University of Melbourne, Melbourne, VIC Australia

**Keywords:** Cardiovascular diseases, Risk factors

## Abstract

**Background:**

The United States has experienced high levels of excess mortality during the COVID-19 pandemic and also has high prevalence of overweight and obesity, which increases the risk of severe infection and death from the virus. This study uses multiple cause of death data to estimate excess premature cardiovascular disease mortality in the USA in 2020 for which overweight and obesity was a risk factor.

**Methods:**

The contribution of overweight and obesity to premature (35–74 years) cardiovascular disease mortality was measured as cardiovascular disease reported on the death certificate with one or more of diabetes, chronic kidney disease, obesity, lipidemias or hypertensive heart disease (DKOLH-CVD). Excess mortality was calculated as the difference between actual and expected age-standardised death rates. Expected deaths were estimated using negative binomial regressions of monthly deaths during 2010–19.

**Results:**

Excess DKOLH-CVD mortality in March-December 2020 was 29% (95% uncertainty interval 28–31%) for males and 30% (28–32%) for females, much higher than for all causes (males 19% (18–21%), females 16% (14–17%)). Excess mortality was higher where two or more DKOLH conditions (males 40% (37–43%), females 41% (37–44%)) or obesity (males 42% (38–45%), females 47% (43–51%)) were reported. One-half of excess DKOLH-CVD mortality was reported as due to COVID-19, lower than the four-fifths of excess all-cause deaths. For home deaths, just over 10% of excess mortality for each cause classification was reported as due to COVID-19.

**Conclusions:**

Excess premature cardiovascular disease mortality in the USA for which overweight and obesity was a risk factor was considerably higher than for all causes, exacerbating adverse pre-pandemic trends. The contribution of COVID-19 to excess mortality appears significantly under-reported for home deaths.

## Background

The COVID-19 pandemic has had a profound impact on mortality in the United States. Estimates of excess mortality—the difference between the actual and expected number of deaths—range from 930,000–1.13 million deaths (or 15–18%) higher than expected for 2020 and 2021 [1–4]. Excess mortality in that period was comprised of an estimated 73–89% of deaths reported as due to COVID-19, depending on the estimate of excess mortality [5]. The remainder of excess mortality would be deaths due to COVID-19 but which were reported as due to another cause, (e.g. no positive SARS-CoV-2 test or inconsistent application of diagnostic criteria for COVID-19 deaths by medical certifiers), deaths that occurred because of the pandemic that were not due to COVID-19 (e.g. people unable to access healthcare because of an overwhelmed health system), deaths averted due to social lockdowns (e.g. from infectious diseases and traffic accidents), and reduced deaths from certain diseases where the person instead died from COVID-19 [3, 4, 6].

There has been considerable epidemiological interest in the contribution of overweight and obesity to excess mortality during the COVID-19 pandemic [7, 8]. Several studies have shown that the risk of severe infection, hospitalisation, intensive care unit admission and death due to COVID-19 is higher for people who have overweight or obesity, especially in non-elderly ages [9–12]. It is likely that overweight and obesity made a significant contribution to excess mortality in the USA because it has the highest prevalence among adults of all high-income countries, it had contributed to slowing mortality declines prior to the pandemic and has also been forecast to adversely affect future life expectancy [13–17].

However, it is challenging to accurately measure the contribution of overweight and obesity to mortality. Medical certifiers have the option of reporting overweight or obesity on a medical certificate of cause of death (or death certificate), but this is under-reported and so does not reflect its true contribution to mortality [18]. The conventional method to measure the population-level contribution of overweight and obesity to mortality is to estimate the population attributable fraction using data from disparate sources: prevalence of high body mass index (BMI) from sample surveys, relative risks of cause-specific mortality due to high BMI compared with a minimum population exposure based on systematic reviews of the literature, and underlying cause of death data [19, 20]. However the population attributable fraction method does not have the flexibility to analyse the contribution of overweight and obesity to mortality according to variables for which BMI prevalence or relative risk data are not available, such as date and place of death.

An alternative is to utilise multiple cause of death data, which comprise all conditions reported on the death certificate. A previous study in the USA and Australia used multiple cause of death data to estimate the contribution of overweight and obesity to cardiovascular disease mortality using deaths where at least one of diabetes, hypertension, chronic kidney disease, lipidemias (high cholesterol) and obesity were reported on the death certificate together with at least one cardiovascular disease (referred to as the acronym *DKOLH-CVD*) [21]. These DKOLH-CVD conditions all share overweight and obesity as a major risk factor, as demonstrated by several studies [19, 22–25]. The above multiple cause of death study found that age-standardised premature (35–74 years) DKOLH-CVD death rates had been increasing in the USA and Australia prior to the pandemic, and that this rise was more pronounced among younger adults where lifetime obesity levels are higher [21].

Multiple cause of death data, which is available for all registered deaths in the USA, can reveal much about the contribution of overweight and obesity to mortality during the COVID-19 pandemic. In particular, it allows for measurement of the contribution of overweight and obesity to mortality using unit record data, and so provides more granular evidence of differentials in the population compared with the population attributable fraction method. This study hence uses multiple cause of death data to estimate excess premature cardiovascular disease mortality (at 35–74 years) for which overweight and obesity was a risk factor, using DKOLH-CVD and measures of more severe overweight/obesity, in the US during the COVID-19 pandemic in 2020 and to assess its contribution to all-cause excess mortality. Excess mortality, and the percentage reported as due to COVID-19, is analysed by sex, age, month of death and place of death, to understand the demographic and temporal variation in these indicators and to compare between hospitals and other places of death (especially in the home) where diagnosis of COVID-19 is more difficult. These analyses include comparison with excess deaths from other causes.

## Data and methods

The study uses unit record data from the Mortality Multiple Cause Files from the National Center for Health Statistics from 2010 to 2020 [26]. Unit record data for 2021 were not available at the time of the analysis. The Mortality Multiple Cause Files provide data on all registered deaths in the USA. The data reports all diseases and conditions reported on the medical certificate of cause of death, which requires the certifier to report the sequence or chain of diseases or conditions leading to death in Part 1 and other significant conditions contributing to death in Part 2 [27]. The data also show the underlying cause of death, which is the disease or injury that initiated the train of morbid events that led to the death [27]. Hence, data from the medical certificate of cause of death can be used to describe complications from and comorbidities with COVID-19 mortality [28].

This study used entity axis data that show the International Classification of Diseases 10th Revision (ICD-10) codes on the death certificate based on how they appeared on the death certificate, consistent with previous application of the DKOLH-CVD measure [21, 27]. The cause classifications used were:

- DKOLH-CVD: Deaths where a cardiovascular disease (ICD-10 codes I00-I99) is mentioned on the death certificate (excluding cardiac arrest (I46) which is a more immediate cause of death where all other reported causes can be unrelated to cardiovascular diseases) along with any of the following: diabetes (E10-E14), chronic kidney diseases (N18), obesity (E65-E66), lipidemias (E78) or hypertensive heart disease (I10-I13).
  - DKOLH2-CVD: The same as DKOLH-CVD, except that two or more of diabetes, chronic kidney diseases, obesity, lipidemias or hypertensive heart disease are reported on the death certificate. This is expected to represent people with higher BMI compared with DKOLH-CVD.
  - Obesity: Deaths where a cardiovascular disease (excluding cardiac arrest) and just obesity are mentioned on the death certificate. This is expected to represent even higher BMI than DKOLH-CVD and DKOLH2-CVD because it is a direct measure of obesity of the deceased individual rather than an indirect estimate of both overweight and obesity.
- Other CVD (i.e. excluding DKOLH-CVD): Deaths where a cardiovascular disease is mentioned on the death certificate, excluding DKOLH-CVD deaths.
- Non-CVD: All deaths excluding those where a cardiovascular disease is mentioned on the death certificate.

COVID-19 was reported in the data as ICD-10 code U07.1. Other variables analysed were age at death, sex of deceased, date of death and place of death (hospital (excluding dead on arrival), home, nursing home/hospice, other). However, no geographic data (e.g. county or state of residence) were available in the dataset and so no spatial differences could be identified, which other studies have found for excess mortality and also longer-term diabetes and chronic kidney disease mortality in the USA [29–31]. The study measured age-standardised death rates for ages 35–74 years using the 2020 US population (both sexes) as the age standard. Population data were obtained from the US Census Bureau [32].

Excess mortality was measured as the actual age-standardised death rate minus the expected age-standardised death rate (absolute) and the actual age-standardised death rate divided by the expected age-standardised death rate minus 1 (relative). The expected age-standardised death rate for each sex was estimated using a negative binomial regression of monthly deaths during 2010–19, with covariates of year of death, a third-degree polynomial of month of death, and five-year age groups (35–39 to 70–74 years), offset by the natural log of population. The negative binomial regression results were used to predict deaths by age and month in 2020, from which age-standardised death rates by month in 2020 and annualised age-standardised death rates for March–December 2020 were calculated (because excess mortality did not occur until March). Regressions were conducted for each combination of sex, cause of death and place of death to calculate expected deaths for each. Uncertainty intervals of the expected age-standardised death rates were calculated based on 1000 simulations of the predicted age-standardised death rate from the negative binomial models while uncertainty intervals of actual age-standardised death rates were calculated assuming a poisson distribution of deaths. Excess mortality with uncertainty was then calculated by conducting 1000 simulations incorporating the uncertainty of both the actual and expected age-standardised death rates.

The proportion of excess mortality due to COVID-19 was calculated, for each cause classification (i.e. DKOLH-CVD etc.), as the age-standardised COVID-19 mortality rate (COVID-19 as the underlying cause of death) divided by the excess age-standardised death rate, with the remainder of excess mortality classified as excess mortality due to non-COVID causes. In some instances, the COVID-19 death rate was higher than the excess death rate and hence mortality due to non-COVID causes was negative (i.e. lower than expected). All analyses were conducted using Stata 16.0 [33]. Code used to generate the results is available by contacting the author.

## Results

Excess premature all-cause mortality in the USA in March–December 2020 was 19% (95% uncertainty interval 18–21%) higher than expected for males and 16% (14–17%) higher for females (Table 1). Excess mortality was considerably higher for DKOLH-CVD, being 29% (28–31%) higher for males and 30% (28–32%) higher for females, and this cause comprised about two-fifths of excess mortality from all causes. Excess mortality was particularly high for DKOLH2-CVD, being 40% (37–43%) for males and 41% (37–44%) for females, and obesity which was 42% (38–45%) higher for males and 47% (43–51%) for females. The lower contribution to all-cause excess mortality by DKOLH2-CVD (males 21%, females 24%) and obesity (males 6%, females 8%) reflects their lower absolute mortality rates. Excess mortality was lower for other CVDs, (males 13% (10–14%), females 8% (6–10%)) and non-CVDs (males 17% (15–18%), females 13% (11–14%)).The extent of excess mortality was approximately proportional to the rate of growth in mortality rates from that cause of death from 2010–19; obesity had the fastest rate of growth during that period, followed by DKOLH2-CVD and DKOLH-CVD, with all-cause mortality rates being stable and non-CVD and other CVD declining slightly (Fig. S1).

**Table 1:** Actual, expected and excess mortality, contribution to all-cause excess mortality and COVID-19 mortality as a fraction of excess mortality (based on age-standardised death rates 35–74 years), by sex and cause of death, USA, March–December 2020.

Category	Actual (per 100,000)	Actual (number of deaths)	Expected (per 100,000)	Excess (%)	Contribution to all-cause excess (%)	COVID-19 DR/excess mortality DR^a^
Male						
All-cause	1154 (1151–1157)	727,734	969 (957–983)	19.1%(17.5–20.8%)	100.0%	77.1% (71.6–83.2%)
DKOLH-CVD	309(308–311)	194,114	239 (234–241)	29.3%(27.6–31.3%)	38.1% (34.8–41.7%)	50.1% (47.5–52.9%)
DKOLH2-CVD^b^	139 (138–140)	87,874	100 (98–102)	39.5% (36.5–42.9%)	21.4% (19.5–23.5%)	53.2%(50.1–56.8%)
Obesity^b^	36 (36–37)	23,221	26 (25–26)	41.5% (37.5–45.4%)	5.8% (5.3–6.4%)	50.8% (47.3–55.1%)
Other CVD (excl. DKOLH-CVD)	244 (243–245)	154,964	217 (214–221)	12.5% (10.5–14.3%)	14.6% (12.1–17.0%)	56.2% (49.8–65.6%)
Non-CVD	606 (604–608)	378,656	518 (509–527)	16.8% (15.4–18.2%)	47.6% (42.5–54.2%)	105.5% (96.4–114.9%)
Female						
All-cause	683 (681–685)	474,331	591 (582–597)	15.9% (14.4–17.4%)	100%	81.0% (75.1–88.0%)
DKOLH-CVD	162 (161–163)	113,283	125 (123–127)	29.5% (27.5–31.7%)	40.3% (36.5–44.5%)	48.5% (45.8–51.6%)
DKOLH2-CVD^b^	77 (76–78)	53,746	55 (54–56)	40.7% (37.4–43.8%)	24.2% (21.8–26.7%)	50.7% (47.6-54.3%)
Obesity^b^	25 (24–25)	16,821	17 (16–17)	46.6% (42.8–50.8%)	8.4% (7.6–9.4%)	51.9% (47.9–56.1%)
Other CVD (excl. DKOLH-CVD)	129 (128–130)	87,507	119 (117–121)	8.0% (6.2–10.0%)	10.3% (8.0–12.8%)	72.1% (63.1–85.5%)
Non-CVD	393 (392–394)	273,541	347 (343–352)	12.9% (11.4–14.4%)	49.9% (42.7–57.2%)	108.7% (97.6–122.9%)

95% uncertainty intervals shown in brackets.

*DR* death rate, *Excl.* excluding.

^a^COVID death rate divided by excess death rate. A figure greater than 100% indicates that the COVID death rate exceeds the excess death rate. A negative figure indicates that the excess death rate was negative.

^b^A subset of DKOLH-CVD.

**Fig. 1:**
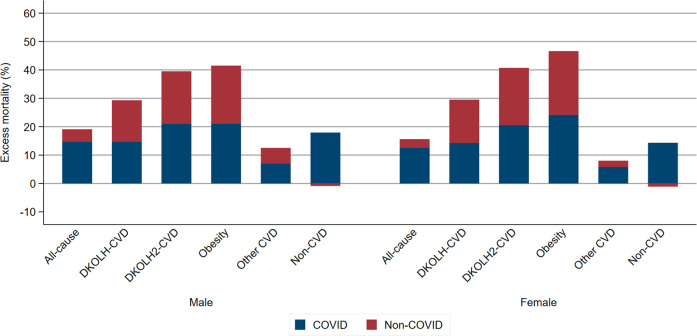
Excess mortality (%, based on age-standardised death rate 35–74 years), and contribution of COVID-19 to excess mortality, by sex and cause of death, USA, March–December 2020. The COVID-19 contribution to excess mortality (blue shaded) was calculated as: (COVID-19 death rate/excess death rate) * excess mortality %. The difference between the blue shaded part and the excess mortality % is the non-COVID contribution to excess mortality (red shaded). The COVID-19 death rate can exceed the excess death rate if there were less deaths than expected from non-COVID causes. In this instance, non-COVID makes a net negative contribution to excess mortality and so is shown as a negative contribution.

COVID-19 comprised 77% of male and 81% of female excess premature mortality for all causes during March–December 2020 (Fig. 1). In contrast, for DKOLH-CVD, DKOLH2-CVD and obesity, COVID-19 death rates were only about one-half of excess mortality. For other CVD deaths, most excess mortality was due to COVID-19, however for non-CVD deaths COVID-19 death rates were slightly higher than excess mortality, resulting in a small decline in non-COVID mortality within this cause of death.

**Fig. 2:**
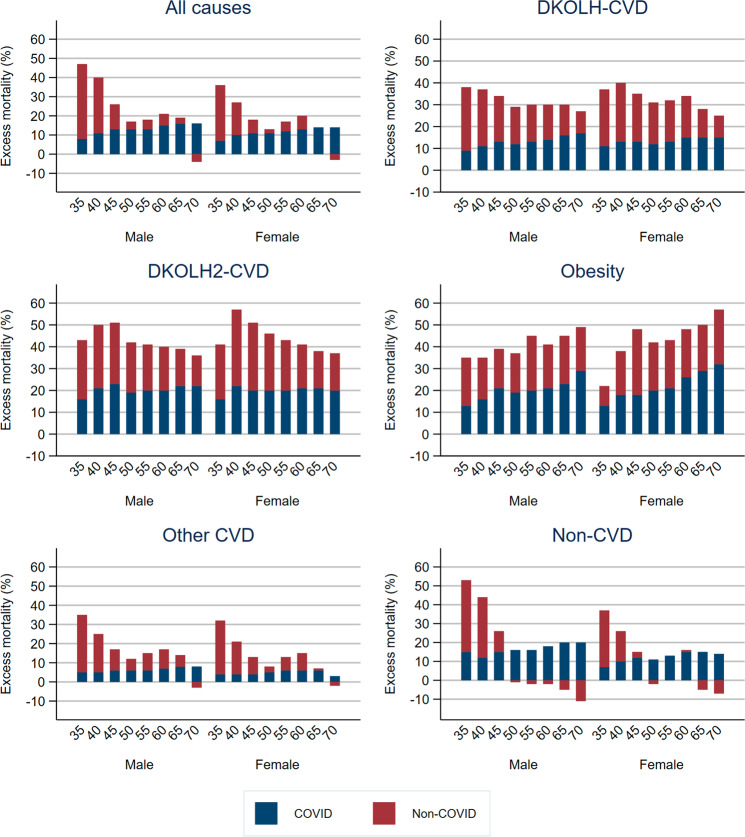
Excess mortality (%, based on age-specific death rates within 35–74 years), and contribution of COVID-19 to excess mortality, by sex, cause of death and age group, USA, March-December 2020. Age refers to start of five-year age group. Uncertainty intervals shown in Table S1. See Fig. 1 for an explanation of negative non-COVID values.

Excess mortality for DKOLH-CVD was highest at ages less than 45 years (just less than 40%), and declined gradually to 70–74 years (just over 25%) (Fig. 2). For DKOLH2-CVD there was also a decline from a peak of over 50% in ages 40–44 years to less than 40% in 70–74 years. Excess obesity mortality, in contrast, increased with age to reach 49% at 70–74 years for males and 57% for females. For all causes, other CVD and non-CVD mortality, excess mortality had a strong negative relationship with age. For example, for all causes excess mortality was 47% at age 35–39 years and just 12% for 70–74 years. For DKOLH-CVD, DKOLH2-CVD and obesity, the COVID-19 death rate comprised a slightly higher fraction of excess mortality as age increased. However, the age gradient was much stronger for all causes, other CVDs and non-CVD excess mortality, leading to the COVID-19 death rate being higher than excess mortality in the oldest ages and non-COVID-19 deaths being lower compared with expected. DKOLH-CVD, DKOLH2-CVD and especially obesity had consistently higher relative excess mortality than for all causes at each month, with DKOLH-CVD peaking at 48% in December, while DKOLH2-CVD and obesity had very high peaks in July and November and again particularly December (DKOLH2-CVD: male 64%, females 63%, obesity: males 72%, females 75%) (Fig. S2).

**Fig. 3:**
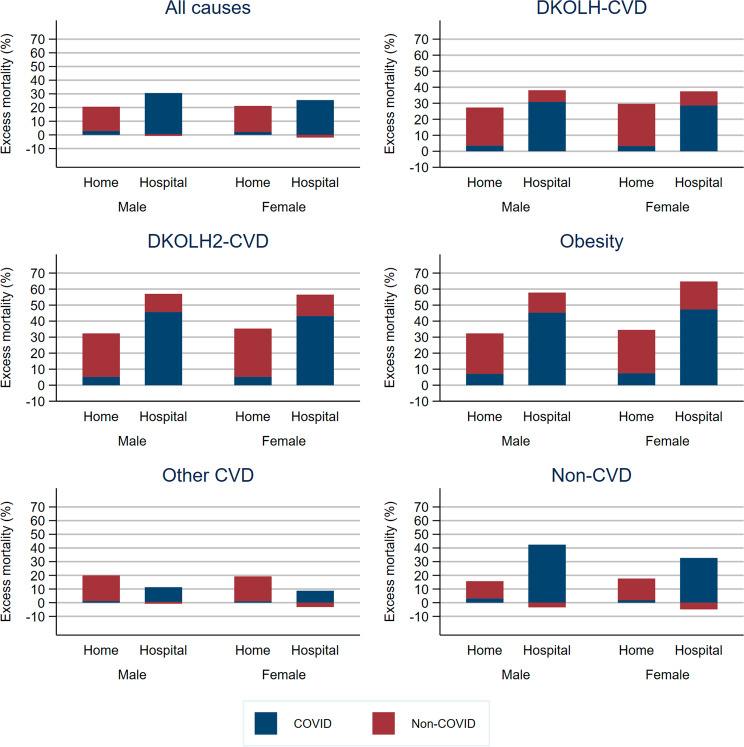
Excess mortality (%, based on age-standardised death rate 35–74 years), and contribution of COVID-19 to excess mortality, by sex, place of death (home and hospital) and cause of death, USA, March–December 2020. See Fig. 1 for an explanation of negative non-COVID values. See Table S3 for uncertainty intervals.

Excess DKOLH-CVD mortality was higher in hospitals (males 38% (35–41%), females 37% (34–41%)) than in the home (males 27% (25–30%), females 30% (27–33%)) (Fig. 3, Table S3). These two places of death are where over four-fifths of deaths occurred in 2020, with the age and sex composition of deaths in each being very similar and the main cause difference being that hospitals had a higher proportion of deaths from chronic kidney disease and a lower proportion of hypertensive heart disease deaths compared with home deaths (Table S4). Excess mortality in the home was slightly higher for DKOLH2-CVD and obesity (DKOLH2-CVD: males 32% (28–37%), females 35% (30–40%); obesity: 32% (27–38%), females 35% (32–39%)), but much higher in hospitals (DKOLH2-CVD: males 57% (52–62%), females 56% (51–62%); obesity: males 58% (52–64%), females 65% (57–72%)). Excess all-cause mortality was also higher in hospitals than in homes, but to a lesser extent. COVID-19 mortality comprised about three-quarters of excess mortality for hospital deaths of DKOLH-CVD, DKOLH2-CVD and obesity, but in the home just over 10% of excess mortality from DKOLH-CVD and DKOLH2-CVD and just over one-fifth of obesity. In the less common place of death of nursing homes/hospices, there was only a slight increase in mortality compared with that which was expected for DKOLH-CVD, DKOLH2-CVD and obesity, with COVID-19 mortality rates being greater than excess mortality, while mortality in other places of deaths exhibited similar characteristics to home deaths.

## Discussion

The impact of the COVID-19 pandemic in the USA was particularly profound for cardiovascular disease mortality of people aged 35–74 years for which overweight and obesity was a risk factor. Excess DKOLH-CVD premature mortality was 30% in March-December 2020, much higher than that for other causes, while it was even higher for DKOLH2-CVD and obesity (reaching over 60% and 70% in December, respectively). This supports previous research showing higher risk of COVID-19 mortality for overweight and obesity [10]. The relative level of cause-specific excess mortality was directly related to their respective rate of increase in recent years; that is, obesity had the highest excess mortality and also had the largest rate of increase in mortality since 2010. Therefore, the COVID-19 pandemic greatly exacerbated the already adverse trends over the previous decade in the USA in premature cardiovascular disease mortality for which overweight and obesity was a risk factor [21].

Only about half of excess premature cardiovascular disease mortality for which overweight and obesity was a risk factor could be attributed to reported COVID-19, compared with about four-fifths for other causes. The major contribution to this difference was that the reported COVID-19 death rate was about 80% of excess DKOLH-CVD mortality in hospitals compared with more than 100% for all causes, while the respective figures for home deaths were just over 10%. The figures for hospital deaths are likely to be far more accurate than for home deaths because of much better testing and diagnosis of COVID-19. Another reason for the lower proportion of excess home deaths being due to COVID-19 could be that over-burdened hospitals were unable to accept some non-COVID patients and many people avoided hospitals due to concerns of being infected, and hence they died at home. This may also be reflected by non-COVID deaths also making a net negative contribution to excess hospital mortality for all causes and some specific cause classifications. However, the absolute net negative contribution of non-COVID deaths to excess mortality from all causes in hospitals was only a very small proportion of the absolute positive contribution of non-COVID deaths to excess home deaths (based on data in Table S3). Hence, under-reporting of COVID-19 mortality is the main reason why it comprised a lower proportion of excess home than hospital deaths. Given that this makes measurement of the exact proportion of excess mortality due to COVID-19 difficult to ascertain, further research is needed to better understand the contributors to excess mortality.

A limitation of this study is that it uses a group of conditions (DKOLH-CVD) to measure cardiovascular disease mortality for which overweight and obesity was a risk factor, without having direct evidence of the BMI of the deceased individuals. However, in the absence of other evidence, the identification of conditions in multiple cause of death data for which overweight and obesity are major risk factors can be used to estimate its contribution to premature cardiovascular disease mortality. The Global Burden of Disease Study estimates that 81% of all deaths at 35–74 years in the US attributed to overweight and obesity have an underlying cause of either diabetes, chronic kidney disease, or cardiovascular diseases (which includes hypertensive heart disease) [25]. Further, diabetes, hypertension and chronic kidney disease have all been identified as increasing the risk of mortality of people with COVID-19, consistent with studies for overweight and obesity and which was also found in our study [10, 34]. Additionally, one study found that 73% of all COVID-19 decedents in US hospitals during March–December 2020 had overweight or obesity, which is similar to the equivalent figure of 67% using this study’s dataset (i.e. for hospital deaths, DKOLH-CVD as a percentage of COVID-19 underlying cause deaths where cardiovascular disease was reported), suggesting that our study estimates the contribution of overweight and obesity to mortality well [10]. The similar age-sex-month-place of death characteristics of excess mortality from DKOLH-CVD and DKOLH2-CVD with reported obesity, aside from the expected different levels, also lend weight to DKOLH-CVD providing plausible estimates of excess premature cardiovascular disease mortality for which overweight and obesity was a risk factor. Although obesity itself is under-reported on death certificates, its higher excess mortality than DKOLH-CVD is as expected given existing evidence of higher BMI and COVID-19 mortality [10, 18].

A major advantage of this study’s approach is that by using data of all registered deaths it allows for granular analyses by conditions, age, sex, month and place of death which provide insights into the differentials in excess mortality by cause group and various characteristics. This method overcomes the use of indirect population attributable fractions, which would be challenging to measure for this study because of not having reliable and accessible BMI data by place of death for 2020. Another advantage of multiple cause data is that it includes all deaths, unlike other analyses which have focused on hospital deaths only [10]. Finally, the expected deaths have relatively low uncertainty intervals because historical trends in mortality in the USA are relatively stable due to the size of the population.

The extent of excess premature cardiovascular disease mortality in the USA in 2020 for which overweight and obesity was a risk factor was immense by any historical comparison, and was likely similar in 2021 based on other estimates of overall excess mortality [9]. Adverse mortality trends from this risk factor were contributing to the slowdown in US life expectancy growth prior to the pandemic and will likely continue to also threaten future longevity growth too in the absence of more concerted public health efforts to reduce obesity, even without considering the possible impact of Long COVID or further significant COVID-19 mortality in coming years [14, 35].

## Supplementary information

Supplementary material

## Data Availability

The datasets generated during and/or analysed during the current study are available from the US National Center for Health Statistics Multiple Cause of Death Data files: https://www.cdc.gov/nchs/data_access/vitalstatsonline.htm#Mortality_Multiple.
