# Supplementary material for: Premature cardiovascular disease mortality with overweight and obesity as a risk factor: estimating excess mortality in the United States during the COVID-19 pandemic
Source: Int J Obes (Lond). 2023 Feb 2;47(4):273–9. doi: 10.1038/s41366-023-01263-y (PMC9891892; doi:10.1038/s41366-023-01263-y)
Supplement: Supplementary file 1 — Supplementary material [file 41366_2023_1263_MOESM1_ESM.docx]

**Supplementary files**

**Premature cardiovascular disease mortality with overweight and obesity as a risk factor: Estimating excess mortality in the United States during the COVID-19 pandemic**

**Tim Adair**

**Figure S1: Trend in cause-specific age-standardised death rate 35-74 years, by sex, US, 2010-2019 and March-December 2020**


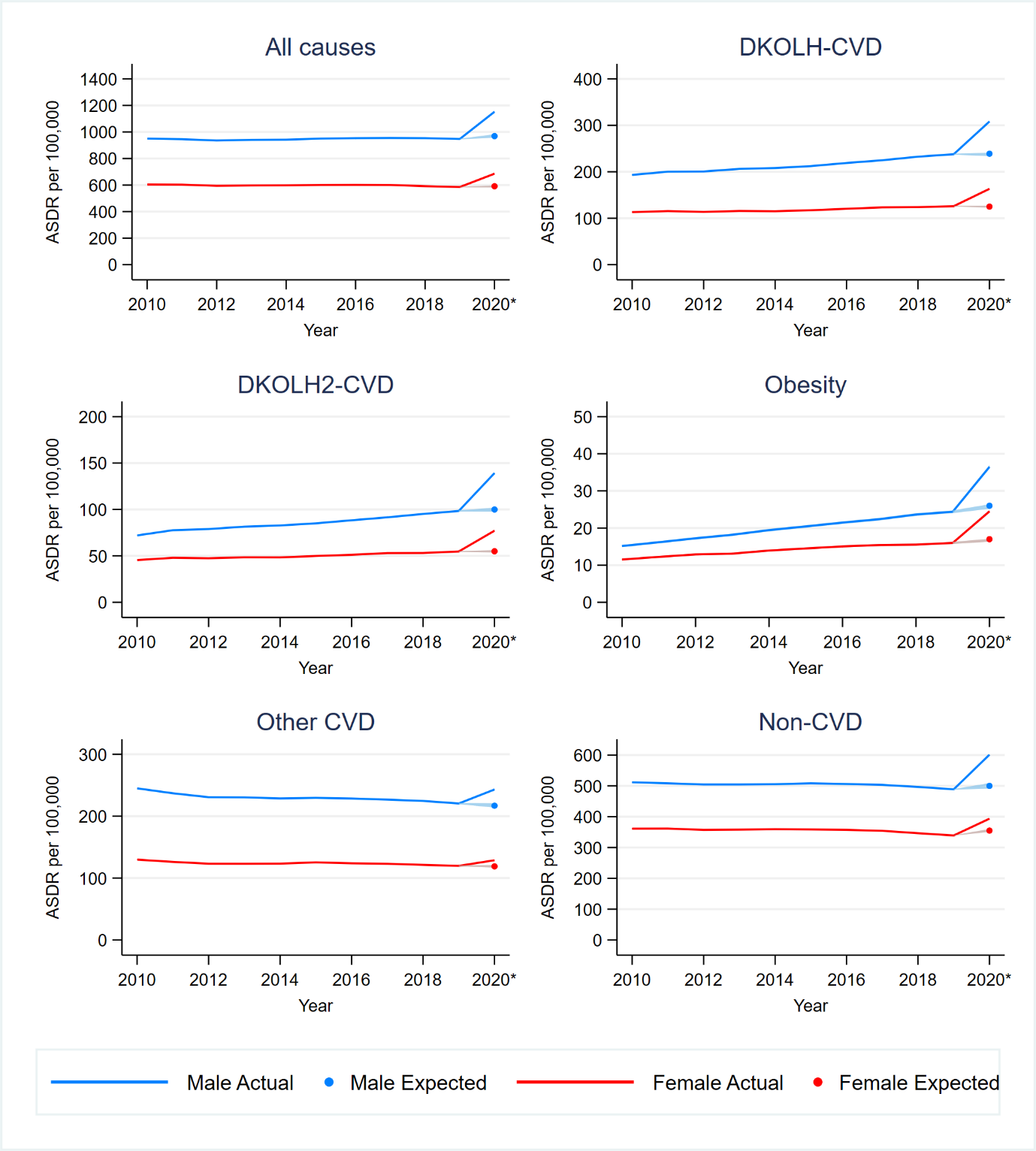


* March-December 2020. ASDR: Age-standardised death rate. Shaded area is 95% uncertainty interval.

**Figure S2: Excess mortality (%, based on age-standardised death rate 35-74 years), by sex, month of death and cause of death, US, March-December 2020**


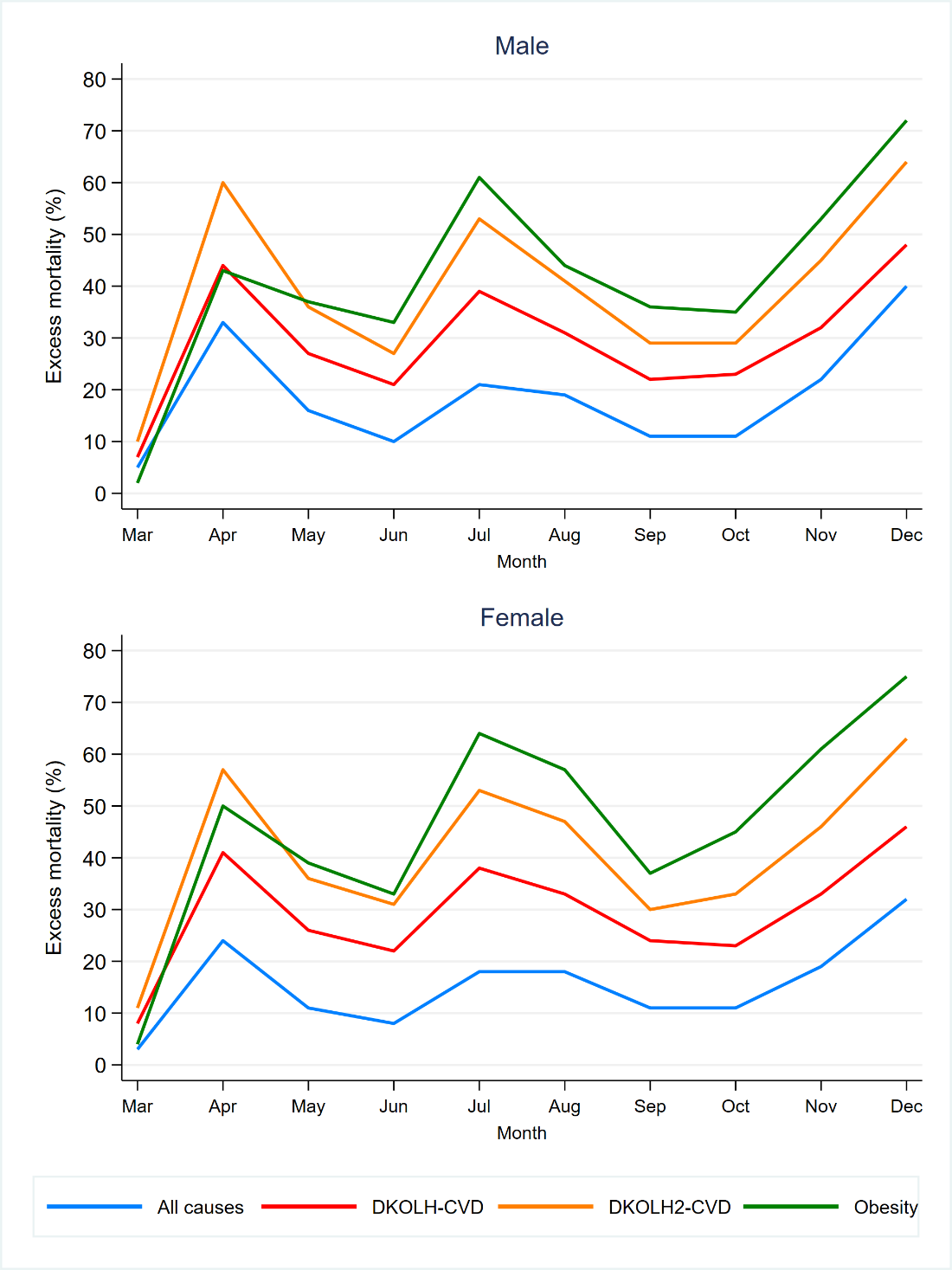


Table S2 shows these results with uncertainty intervals.

**Table S1: Excess mortality (%, based on age-standardised death rate 35-74 years), by sex, age group and cause of death, US, March-December 2020**

| **Age** | **All-cause** | **DKOLH-CVD** | **DKOLH2-CVD** | **Obesity** | **Other CVDs** | **Non-CVD** |
| --- | --- | --- | --- | --- | --- | --- |
| **Male** | | | | | | |
| 35-39 | 47.2  (43.0-52.2) | 38.5  (31.7-44.9) | 42.5  (32.1-53.4) | 35.0  (24.0-47.7) | 35.2  (28.8-41.9) | 53.0  (47.5-58.9) |
| 40-44 | 40.2  (35.7-44.8) | 37.2  (31.4-42.8) | 50.4  (40.9-60.2) | 34.4  (24.2-45.3) | 24.4  (18.4-30.2) | 44.4  (38.9-49.6) |
| 45-49 | 26.3  (22.3-30.2) | 33.8  (29.0-39.4) | 50.3  (42.4-58.4) | 39.4  (29.4-50.5) | 16.1  (11.3-21.0) | 26.6  (22.4-30.8) |
| 50-54 | 17.5  (14.0-21.1) | 29.1  (24.6-33.9) | 42.1  (34.7-49.6) | 37.0  (26.9-48.0) | 12.4  (8.1-16.8) | 15.2  (11.5-18.8) |
| 55-59 | 17.7  (13.9-21.7) | 30  (25.5-34.5) | 41.2  (35.0-47.9) | 44.3  (34.5-55.4) | 14.8  (10.4-19.1) | 13.5  (9.8-17) |
| 60-64 | 20.1  (16.4-23.9) | 30.1  (25.9-34.5) | 39.3  (32.8-46.5) | 40.5  (31.4-50.7) | 17.4  (12.9-21.9) | 15.8  (12.4-19.3) |
| 65-69 | 18.7  (15.1-22.6) | 29.9  (25.7-34.4) | 38.2  (31.5-44.8) | 44.3  (34.2-54.2) | 13.2  (9.3-17.1) | 15.2  (10.8-19.2) |
| 70-74 | 12.4  (8.9-15.8) | 26.6  (22.4-31.0) | 36.8  (30.8-43.0) | 48.4  (37.8-60.6) | 5.3  (1.4-9.2) | 8.1  (4.2-11.1) |
| **Female** | | | | | | |
| 35-39 | 35.8  (31.0-40.5) | 36.7  (29.5-45.3) | 42  (29.6-55.0) | 22.4  (10.1-35.4) | 32.5  (24.8-40.7) | 37.2  (32.8-41.6) |
| 40-44 | 26.7  (22.1-31.1) | 39.5  (31.4-46.3) | 56.8  (45.7-68.5) | 37.4  (25.7-50.5) | 21.4  (14.8-28.2) | 26.3  (22.0-30.5) |
| 45-49 | 17.6  (13.8-21.4) | 34.9  (29.3-41.1) | 51.4  (42.0-60.9) | 47.4  (35.2-61.0) | 13.0  (7.3-18.5) | 14.9  (11.3-18.3) |
| 50-54 | 13.0  (9.7-16.8) | 31.2  (26.1-37.0) | 46.3  (38.3-55.5) | 41.8  (31.7-53.3) | 7.8  (3.3-12.8) | 9.4  (5.9-12.8) |
| 55-59 | 16.7  (13.3-20.3) | 32.3  (27.5-37) | 42.3  (34.6-50.4) | 43.3  (33.1-54.8) | 12.8  (8.2-17.6) | 13.1  (9.6-17.2) |
| 60-64 | 19.9  (16.3-23.3) | 33.9  (29.0-39.0) | 41  (34.1-48.0) | 48.3  (38.1-59.6) | 14.3  (10.1-18.7) | 16.2  (12.7-19.7) |
| 65-69 | 14.0  (10.7-17.3) | 27.9  (23.7-32.1) | 38.4  (32.2-44.9) | 50.6  (41.0-60.5) | 7.1  (3.2-11.4) | 10.4  (6.7-14.2) |
| 70-74 | 11.1  (7.9-14.2) | 25.2  (20.8-29.7) | 37.9  (31.0-44.9) | 57.0  (45.1-69.6) | 1.1  (-2.4-4.7) | 6.9  (2.1-11.3) |

**Table S2: Excess mortality (%, based on age-standardised death rate 35-74 years), by sex, month of death and cause of death, US, March-December 2020**

| **Month** | **All-cause** | **DKOLH-CVD** | **DKOLH2-CVD** | **Obesity** |
| --- | --- | --- | --- | --- |
| **Male** | | | | |
| March | 5.0  (0.5-9.7) | 7.5  (2.6-12.6) | 9.7  (2.2-17.5) | 2.0  (-5.9-11.3) |
| April | 32.7  (27.1-38.3) | 43.7  (37.0-50.9) | 59.9  (49.0-71.2) | 43.6  (31.9-55.6) |
| May | 16.0  (11.4-20.6) | 26.5  (20.1-32.5) | 36.1  (27.4-45.1) | 37.2  (24.9-50.3) |
| June | 10.1  (5.6-15.0) | 21.3  (15.2-27.4) | 26.6  (17.9-34.4) | 32.2  (20.6-44.3) |
| July | 21.3  (16.3-26.3) | 38.9  (32.5-45.4) | 52.6  (42.7-63.5) | 60.5  (49.1-75.8) |
| August | 18.6  (13.7-23.3) | 31  (24.8-37.5) | 41.3  (31.4-51.5) | 43.7  (32.0-56.3) |
| September | 11.5  (6.9-15.8) | 22.3  (16.7-28.5) | 28.5  (19.7-37.6) | 35.8  (23.9-49.5) |
| October | 11.4  (7.4-15.7) | 22.7  (16.7-28.9) | 29.4  (20.6-38.5) | 35.4  (24.9-48.3) |
| November | 22.4  (18.2-26.9) | 32.1  (26.5-38.5) | 45.4  (36.5-56.3) | 53.4  (39.9-67.9) |
| December | 40.3  (34.9-46.4) | 47.7  (40.6-54.5) | 64.5  (54.5-75.4) | 71.2  (57.2-85.8) |
| **Female** | | | | |
| March | 3.2  (-0.7-7.4) | 8.2  (2.6-13.7) | 11.2  (3.4-19.3) | 3.8  (-5.9-13.7) |
| April | 23.9  (19.0-29.1) | 41.5  (33.9-49.4) | 56.6  (45.5-69.7) | 50.1  (36.7-64.5) |
| May | 11.0  (6.6-15.7) | 26.1  (19.6-32.5) | 36.3  (26.8-46) | 39.3  (27.1-52.3) |
| June | 7.7  (3.4-12.2) | 22.4  (16.5-28.7) | 30.5  (20.8-39.9) | 33.2  (20.9-46.6) |
| July | 18.2  (13.4-22.9) | 37.7  (30.8-44.3) | 52.9  (42.7-64) | 64.5  (50.6-80.8) |
| August | 17.6  (12.7-22.7) | 32.8  (26.2-39.1) | 47.1  (36.9-57.4) | 57  (43-72.5) |
| September | 11.0  (6.2-15.8) | 23.9  (17.7-30.3) | 29.5  (20.5-39.4) | 37.3  (24.7-51.9) |
| October | 11.4  (7.1-15.6) | 23.3  (17.0-30.5) | 33.2  (23.1-42.7) | 44.5  (31.9-59.1) |
| November | 19.2  (14.1-24.3) | 33.4  (26.9-40.2) | 46  (35.6-56.8) | 60.6  (46.8-75.8) |
| December | 32.4  (27.1-37.8) | 46.2  (38.7-53.7) | 62.6  (51.3-74.4) | 74.3  (59.1-90.8) |

95% uncertainty intervals shown in brackets

**Table S3: Actual, expected and excess mortality and COVID-19 mortality as a fraction of excess mortality (based on age-standardised death rate 35-74 years), by sex, place of death and cause of death, US, March-December 2020**

|  | **Male** | | | | **Female** | | | |
| --- | --- | --- | --- | --- | --- | --- | --- | --- |
| **Place of death / Category** | **Actual**  **(per 100,000)** | **Expected**  **(per 100,000)** | **Excess (%)** | **COVID-19 DR / Excess mortality DR*** | **Actual**  **(per 100,000)** | **Expected**  **(per 100,000)** | **Excess (%)** | **COVID-19 DR / Excess mortality DR*** |
| **Home** | | | | | | | | |
| All-cause | 420  (418-421) | 347  (343-352) | 21% (19-22%) | 14% (13-15%) | 247  (246-248) | 204  (201-207) | 21% (19-23%) | 10% (9-11%) |
| DKOLH-CVD | 132  (131-133) | 104  (102-106) | 27% (25-30%) | 13% (12-15%) | 66 (65-66) | 50 (49-51) | 30% (27-33%) | 11% (10-12%) |
| DKOLH2-CVD** | 60 (59-61) | 45 (44-47) | 32% (28-37%) | 16% (14-18%) | 31 (30-31) | 23 (22-23) | 35% (30-40%) | 15% (13-16%) |
| Obesity** | 17 (17-18) | 13 (13-14) | 32% (27-38%) | 22% (19-26%) | 10 (10-11) | 8 (7-8) | 35% (32-39%) | 22% (19-26%) |
| Other CVDs | 77 (76-77) | 64 (62-66) | 20% (17-23%) | 6% (5-7%) | 37 (36-37) | 31 (30-32) | 19% (16-23%) | 6% (5-7%) |
| Non-CVDs | 210  (208-211) | 181  (179-183) | 16% (14-18%) | 20% (19-22%) | 145  (144-146) | 123  (122-125) | 18% (16-20%) | 10% (9-11%) |
| **Hospital** | | | | | | | | |
| All-cause | 509  (507-511) | 392  (386-398) | 30% (28-32%) | 101%  (96-107%) | 305  (303-306) | 248  (244-252) | 23% (21-26%) | 108%  (101-116%) |
| DKOLH-CVD | 125  (124-126) | 91 (89-92) | 38% (35-41%) | 81% (76-86%) | 69 (69-70) | 51 (50-52) | 37% (34-41%) | 76% (72-83%) |
| DKOLH2-CVD** | 58 (57-59) | 37 (36-38) | 57% (52-62%) | 80% (76-86%) | 33 (33-34) | 21 (21-22) | 56% (51-62%) | 76% (71-82%) |
| Obesity** | 15 (15-15) | 9 (9-10) | 58% (52-64%) | 78% (72-86%) | 11 (11-12) | 7 (7-7) | 65% (57-72%) | 73% (67-81%) |
| Other CVDs | 126  (126-127) | 114  (112-116) | 11% (9-13%) | 102%  (86-127%) | 69 (69-70) | 66 (65-67) | 5% (3-7%) | 158%  (118-250%) |
| Non-CVDs | 259  (257-360) | 186  (183-188) | 39% (37-41%) | 109%  (103-115%) | 167  (166-168) | 130  (129-132) | 28% (26-30%) | 118%  (108-130%) |
| **Nursing home / Hospice** | | | | | | | | |
| All-cause | 130  (129-131) | 145  (141-149) | -10%  (-13--8%) | -73%  (-101%--57%) | 97 (96-98) | 114  (111-117) | -15%  (-17--12%) | -43%  (-52--36%) |
| DKOLH-CVD | 31 (31-32) | 30 (27-33) | 4% (-9-17%) | 150%  (-3842-3356%) | 21 (21-22) | 21 (19-23) | 3% (-7-14%) | 122%  (-2561-1820%) |
| DKOLH2-CVD** | 14 (13-14) | 13 (12-15) | 4% (-5-14%) | 281%  (-2518-2580%) | 10 (9-10) | 9 (8-11) | 3% (-8-16%) | 282%  (-2390-1627%) |
| Obesity** | 1 (1-1) | 1 (1-1) | 1% (-13-18%) | 1548%***  (-2266-4193%) | 1 (1-2) | 1 (1-2) | 4% (-12-20%) | 438%***  (-3797-2836%) |
| Other CVDs | 26 (26-27) | 29 (28-32) | -11%  (-19--3%) | -48%  (-207--24%) | 18 (17-18) | 20 (18-22) | -12%  (-19--4%) | -38%  (-114--22%) |
| Non-CVDs | 73 (72-73) | 87 (85-88) | -16%  (-18--11%) | -46%  (-56--40%) | 58 (57-58) | 73 (73-74) | -21%  (-23--17%) | -29%  (-34--26%) |
| **Other** | | | | | | | | |
| All-cause | 94 (93-95) | 77 (75-79) | 22% (19-25%) | 11% (10-12%) | 36 (36-36) | 28 (28-29) | 27% (24-31%) | 10% (9-11%) |
| DKOLH-CVD | 20 (20-21) | 17 (16-17) | 23% (17-28%) | 17% (14-22%) | 7 (7-7) | 5 (5-5) | 35% (27-43%) | 14% (11-18%) |
| DKOLH2-CVD** | 8 (8-8) | 6 (6-7) | 28% (19-37%) | 24% (18-33%) | 3 (3-3) | 2 (2-2) | 40% (29-51%) | 24% (17-32%) |
| Obesity** | 3 (3-3) | 3 (2-3) | 18% (8-29%) | 50% (31-104%) | 1 (1-1) | 1 (1-1) | 16% (3-30%) | 96% (44-366%) |
| Other CVDs | 14 (13-14) | 11 (11-12) | 23% (18-28%) | 11% (8-14%) | 5 (5-5) | 4 (3-4) | 32% (24-41%) | 9% (6-14%) |
| Non-CVDs | 60 (60-61) | 49 (48-50) | 22% (18-26%) | 9% (8-11%) | 24 (24-24) | 20 (19-20) | 24% (20-28%) | 10% (9-12%) |

* COVID death rate divided by excess death rate. A figure greater than 100% indicates that the COVID death rate exceeds the excess death rate. A negative figure indicates that the excess death rate was negative.

**Subset of DKOLH-CVD

***This shows that the reported COVID-19 death rate for deaths in nursing homes/hospices with obesity reported on the death certificate is several times higher than the absolute excess mortality from this cause in this place.

DR: death rate

95% uncertainty intervals shown in brackets. Wide uncertainty intervals for COVID-19 DR / excess mortality DR for nursing home/hospice reflect the low excess mortality.

**Table S4: DKOLH-CVD components by sex, age group and cause of death, home and hospital deaths, 35-74 years, US, March-December 2020 (%)**

|  | **Home** | **Hospital** |
| --- | --- | --- |
| **Sex** | | |
| Male | 64.7 | 61.4 |
| Female | 35.3 | 38.7 |
| **Age group** | | |
| 35-39 | 1.5 | 1.8 |
| 40-44 | 2.8 | 3.2 |
| 45-49 | 5.3 | 5.7 |
| 50-54 | 9.6 | 9.3 |
| 55-59 | 15.1 | 14.0 |
| 60-64 | 19.8 | 18.7 |
| 65-69 | 22.1 | 22.4 |
| 70-74 | 23.8 | 24.9 |
| **Cause** | | |
| Diabetes | 41.7 | 42.7 |
| Chronic kidney disease | 11.1 | 23.1 |
| Obesity | 12.8 | 10.9 |
| Lipidemias | 13.3 | 10.4 |
| Hypertensive heart disease | 76.6 | 66.3 |

Note: Each category sums to 100%
